# Supplementary material for: Self-organized patterning of cell morphology via mechanosensitive feedback
Source: eLife. 2021 Mar 26;10:e57964. doi: 10.7554/eLife.57964 (PMC8133777; doi:10.7554/eLife.57964)
Supplement: Transparent reporting form [file elife-57964-transrepform.pdf]

eLIFE
Westbrook Centre, Milton Road
Cambridge CB4 1YG
UK
P 01223 855340
W elifesciences.org
T @elife

eLife's transparent reporting form

We encourage authors to provide detailed information *within their submission* to facilitate the interpretation and replication of experiments. Authors can upload supporting documentation to indicate the use of appropriate reporting guidelines for health-related research (see EQUATOR Network), life science research (see the BioSharing Information Resource), or the ARRIVE guidelines for reporting work involving animal research. Where applicable, authors should refer to any relevant reporting standards documents in this form.

If you have any questions, please consult our Journal Policies and/or contact us: editorial@elifesciences.org.

Sample-size estimation

- You should state whether an appropriate sample size was computed when the study was being designed
- You should state the statistical method of sample size computation and any required assumptions
- If no explicit power analysis was used, you should describe how you decided what sample (replicate) size (number) to use

Please outline where this information can be found within the submission (e.g., sections or figure legends), or explain why this information doesn't apply to your submission:

No explicit sample size estimation was done when the experiment was designed.

The live imaging data are laborious to replicate, given that cell segmentation and tracking require manual proofreading that requires weeks of concentrated effort. We analyzed the first five high-quality movies we acquired, in which the pouch region was visible with minimal photobleaching for 13hrs. This number is reported in the text, figure legend, and methods. We present averaged data in the main figures but also provide the data for individual movies in the corresponding Source Data files (Dryad).

For the genetic perturbation data (Fig 5 and 7), the sample size used for each experiment was generally limited by how many discs could be dissected by hand and imaged during a reasonable time frame (~1hr). The number of samples analyzed for each genotype is reported in the figure legend. The main figures present averaged data, but we also include the data from individual samples in the corresponding Source Data files. All samples were analyzed, except in very rare cases when the discs were improperly mounted and thus not fully visible.

Replicates

- You should report how often each experiment was performed
- You should include a definition of biological versus technical replication
- The data obtained should be provided and sufficient information should be provided to indicate the number of independent biological and/or technical replicates
- If you encountered any outliers, you should describe how these were handled
- Criteria for exclusion/inclusion of data should be clearly stated
- High-throughput sequence data should be uploaded before submission, with a private link for reviewers provided (these are available from both GEO and ArrayExpress)

Please outline where this information can be found within the submission (e.g., sections or figure legends), or explain why this information doesn't apply to your submission:

The live imaging data includes 5 samples, each acquired on separate days. This information is listed in the main text, figure legend and methods sections.

The laser ablation data includes at least 19 samples for each region (away and near to DV boundary), acquired over 5 experiment days. We combined data from all days, as no systematic error related to date of acquisition was found. Detailed description of the criteria for exclusion/inclusion of samples is provided in the methods section, as well as in the legend of Figure 4—Figure Supplement 1. While the main text (Fig 4C) excludes outliers, we include them in Figure 4—Figure Supplement 1. We also supply the data as Source Data Figure 4—Source Data 1.

The genetic perturbation data includes at least 6 biological replicates (different wing discs), all from the same day of acquisition. All samples were analyzed, except in very rare cases when the discs were improperly mounted and thus not fully visible.

Statistical reporting

- Statistical analysis methods should be described and justified
- Raw data should be presented in figures whenever informative to do so (typically when N per group is less than 10)
- For each experiment, you should identify the statistical tests used, exact values of N, definitions of center, methods of multiple test correction, and dispersion and precision measures (e.g., mean, median, SD, SEM, confidence intervals; and, for the major substantive results, a measure of effect size (e.g., Pearson's r, Cohen's d)
- Report exact p-values wherever possible alongside the summary statistics and 95% confidence intervals. These should be reported for all key questions and not only when the p-value is less than 0.05.

Please outline where this information can be found within the submission (e.g., sections or figure legends), or explain why this information doesn't apply to your submission:

Raw data are provided as Source Data files or on Dryad. N for all experiments is listed in the figure legends. No statistical tests were applied.

(For large datasets, or papers with a very large number of statistical tests, you may upload a single table file with tests, Ns, etc., with reference to sections in the manuscript.)

Group allocation

- Indicate how samples were allocated into experimental groups (in the case of clinical studies, please specify allocation to treatment method); if randomization was used, please also state if restricted randomization was applied
- Indicate if masking was used during group allocation, data collection and/or data analysis

Please outline where this information can be found within the submission (e.g., sections or figure legends), or explain why this information doesn't apply to your submission:

Samples were grouped by genotype, as indicated in the figure legends.

Additional data files ("source data")

- We encourage you to upload relevant additional data files, such as numerical data that are represented as a graph in a figure, or as a summary table
- Where provided, these should be in the most useful format, and they can be uploaded as "Source data" files linked to a main figure or table
- Include model definition files including the full list of parameters used
- Include code used for data analysis (e.g., R, MatLab)
- Avoid stating that data files are "available upon request"

Please indicate the figures or tables for which source data files have been provided:

We have included source data files for Figures 1H-M, 2, 4, 5, and 7. The data on cell area and elongation in Figure 1A-F, 3F,G, and 6C are too large to be submitted here and have been uploaded to Dryad. Model fit parameters are listed in Table 1 and Appendix 1.
